# Forecasting Root-Zone Electrical Conductivity of Nutrient Solutions in Closed-Loop Soilless Cultures via a Recurrent Neural Network Using Environmental and Cultivation Information

**DOI:** 10.3389/fpls.2018.00859

**Published:** 2018-06-21

**Authors:** Taewon Moon, Tae In Ahn, Jung Eek Son

**Affiliations:** Department of Plant Science, Research Institute of Agriculture and Life Sciences, Seoul National University, Seoul, South Korea

**Keywords:** black box modeling, environmental factor, long short-term memory, machine learning, sweet pepper

## Abstract

In existing closed-loop soilless cultures, nutrient solutions are controlled by the electrical conductivity (EC) of the solution. However, the EC of nutrient solutions is affected by both growth environments and crop growth, so it is hard to predict the EC of nutrient solution. The objective of this study was to predict the EC of root-zone nutrient solutions in closed-loop soilless cultures using recurrent neural network (RNN). In a test greenhouse with sweet peppers (*Capsicum annuum* L.), data were measured every 10 s from October 15 to December 31, 2014. Mean values for every hour were analyzed. Validation accuracy (*R*^2^) of a single-layer long short-term memory (LSTM) was 0.92 and root-mean-square error (RMSE) was 0.07, which were the best results among the different RNNs. The trained LSTM predicted the substrate EC accurately at all ranges. Test accuracy (*R*^2^) was 0.72 and RMSE was 0.08, which were lower than values for the validation. Deep learning algorithms were more accurate when more data were added for training. The addition of other environmental factors or plant growth data would improve model robustness. A trained LSTM can control the nutrient solutions in closed-loop soilless cultures based on predicted future EC. Therefore, the algorithm can make a planned management of nutrient solutions possible, reducing resource waste.

## Introduction

Due to benefits including improved crop yield and quality, soilless cultures in greenhouses have been growing rapidly in popularity. However, most open-loop soilless cultures release drainage nutrient solutions without treatment, causing environmental pollution such as eutrophication and accumulation of heavy metals (Fargašová, 1994; Siddiqi et al., 1998; Le Bot et al., 2001; Nicoletto et al., 2017). To resolve this problem, closed-loop soilless cultures are being studied as sustainable crop cultivation systems. In commercialized closed-loop soilless cultures, nutrient solutions are controlled based on electrical conductivity (EC) because it is easily measured by sensors. Since EC of solutions shows a linear relationship with total equivalents of ions in solutions (Griffin and Jurinak, 1973), EC-based systems have been used to control nutrient solution supply.

In soilless culture systems, root-zone EC should be controlled within target range because it significantly influences the growth and quality of crops (Sonneveld and Voogt, 2009). In general, root-zone EC dynamically varies due to environmental changes and can be controlled by adjusting the concentration of nutrient solutions. In open-loop soilless culture, these EC control processes only consider the resource usage of the system (Ku and Hershey, 1991). However, in closed-loop soilless culture, the discharge of drainage is restricted. Therefore, changes in the EC and the drainage amount are directly affected by the available concentration range of supplying nutrient solutions and the amounts of water and stock solutions for replenishment (Savvas and Manos, 1999). In the EC-based closed-loop soilless culture, which conducts minimal nutrient calibration with EC, these features may affect the stability of nutrient control (Savvas and Manos, 1999; Savvas, 2002; Massa et al., 2011). In order to maintain system reliability under these limited conditions, current control processes should be determined based on the prediction of future changes, which requires an appropriate predictive model (Draeger et al., 1995). Therefore, predicting EC is important for nutrient management of closed soilless cultures.

Although various prediction methods have been developed, nutrient control systems are usually based on contemporary EC monitoring and are vulnerable to ion balance in root-zone nutrient solutions (Neto et al., 2014; Kinoshita et al., 2016). These limits result from crops influencing changes in EC and from growth environments (Dewir et al., 2005; Stutte, 2006; Shin and Son, 2016). Because root-zone nutrient solutions are affected by environmental changes within greenhouses, predicting future changes in the EC of root-zone nutrient solutions is not easy. Prediction of EC needs various environmental data and system parameters; however, EC is greatly affected by environments in a wide range of climate changes (Savvas and Manos, 1999; Savvas, 2002; Lykas et al., 2006; Massa et al., 2011; Shin et al., 2016). It is difficult to apply a control system developed in a specific region to another region of different climate conditions. In fact, no studies have attempted to predict and forecast future EC in various climate conditions.

Deep learning has been used to draw meaningful interpretations from complicated nonlinear data (Mnih et al., 2015; Silver et al., 2016). Deep learning can be used for high-level abstraction from raw data (LeCun et al., 2015). As a part of deep learning, recurrent neural networks (RNNs) are used to analyze chronological data such as for voice and video recognition and natural language processing; this method shows better accuracy than previous algorithms (Adavanne et al., 2017; Ororbia et al., 2017).

Recurrent neural network has an advantage of inputting big data of relatively long period and the length of output values is also unlimited theoretically (Hochreiter and Schmidhuber, 1997). EC in soilless culture is a chronological factor which is difficult to predict because the future EC changes are affected by the accumulation data of past environments and plant growth. To improve EC-based nutrient controls in various climate conditions, prediction of EC should be conducted based on previous environmental factors in closed-loop soilless culture systems. The objective of this study was to predict the EC of root-zone nutrient solutions in closed-loop soilless cultures using RNN algorithms.

## Materials and Methods

### Cultivation Conditions

**FIGURE 1:**
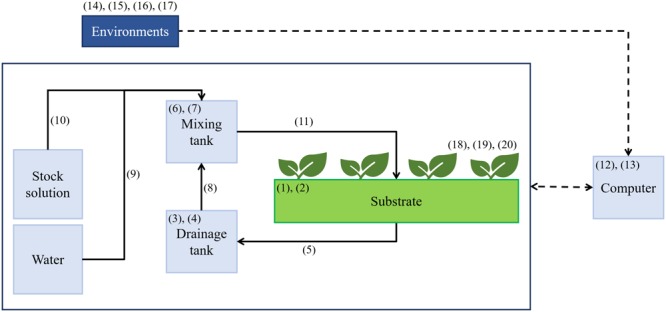
A diagram of a closed-loop soilless culture system and measured data of nutrient solutions and growth environments. Refer to **Table 1** for the measured data (1–20).

**FIGURE 2:**
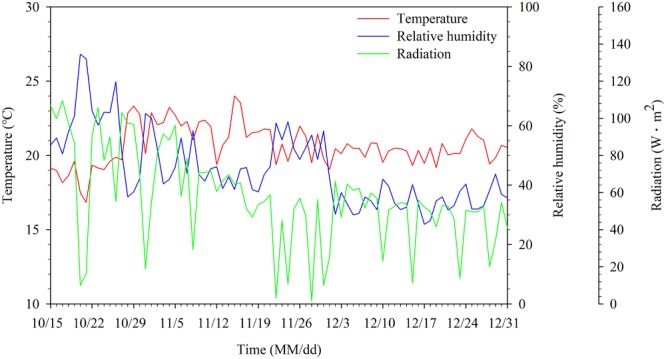
Daily averages of temperature, relative humidity, and radiation in the greenhouse from 15 October to 31 December. Zeros were excluded when radiation was averaged.

A Venlo-type greenhouse at the experimental farm of Seoul National University, Suwon, Korea (37.3° N 127.0° E) was used for experiments. Three sweet pepper (*Capsicum annuum* L.) plants were grown in a rockwool slab and seven slabs were used per row. In this study, four cultivation lines were installed in the greenhouse, each of which is an independent closed-loop soilless culture system having mixing tank, drainage tank, and stock solutions (**Figure 1**). The stock solution was divided into A and B based on the PBG nutrient solution of Netherlands. One of the cultivation lines was used for the experiment. In the greenhouse, daytime temperature was maintained at 25–35°C and nighttime temperature at 17–22°C (**Figure 2**). Outside temperature during the experiment was at -10.8–23°C. EC of nutrient solutions was maintained at 2.6–3.0 dS⋅m^-1^ and pH at 5.5–6.5. Integrated solar radiation method was applied for irrigation control. Nutrient solutions’ composition was 14.17 meq⋅L^-1^ of ${\mathrm{NO}}_{3}^{–}$, 1.14 meq⋅L^-1^ of H_2_${\mathrm{PO}}_{4}^{–}$, 5.92 meq⋅L^-1^ of K^+^, 8.85 meq⋅L^-1^ of Ca^2+^, 3.17 meq⋅L^-1^ of Mg^2+^, and 3.20 meq⋅L^-1^ of ${\mathrm{SO}}_{4}^{2–}$ as macro elements; and 0.038 meq⋅L^-1^ of Fe^2+^, 0.020 meq⋅L^-1^ of Zn^2+^, 0.003 meq⋅L^-1^ of Cu^2+^, 0.021 meq⋅L^-1^ of Mn^2+^, and 0.001 meq⋅L^-1^ of ${\mathrm{MoO}}_{4}^{2–}$ as micro elements. After irrigation event, the drainage was returned to the reservoir tank (52 cm × 26 cm × 26 cm). EC and pH in the reservoir tanks were monitored every 3 days by using a multimeter (Multi 3420 SET C, WTW, Germany). EC and water content in the root media were measured by using a TDR sensor (Grodan, WCM-control, Denmark). EC and pH of fresh water were 0.17 dS⋅m^-1^ and 7.11, respectively, containing 0.21 meq⋅L^-1^ of Na^2+^, 0.29 meq⋅L^-1^ of Cl^-^, 0.04 meq⋅L^-1^ of K^+^, 0.71 meq⋅L^-1^ of Ca^2+^, 0.21 meq⋅L^-1^ of Mg^2+^, 0.19 meq⋅L^-1^ of ${\mathrm{SO}}_{4}^{2–}$, 0.39 meq⋅L^-1^ of ${\mathrm{NO}}_{3}^{–}$, and 0.04 meq⋅L^-1^ of ${\mathrm{PO}}_{4}^{3–}$. Drainage ratios were maintained at 20–30% during experimental period. Plants were grown to maintain two main stems, which were vertically trellized to a “V” canopy system (Jovicich et al., 2004).

### Data Collection

Data on nutrient solutions and growth environments were measured to train the algorithm (**Table 1**). The ECs of nutrient solutions in the mixing tank and drainage tank were measured by EC sensors (SCF-01A, DIK, Korea). The EC and moisture content of substrates were measured by a FDR sensor (CoCo 100B, Mirae Sensor, Korea). CO_2_ concentration and light intensity in the greenhouse were measured by using a nondispersive infrared CO_2_ sensor (KCD-AN300, Sensecube, Korea) and by a pyranometer (SP-110, Apogee, United States), respectively. Data were measured every 10 s from October 15 to December 31, 2014. Mean values for every hour were used. A total of 1,416 data points was used for this study.

**Table 1:** Ranges of measured input data in closed-loop soilless cultures.

(Number) Input data (unit)	Range
(1) Electrical conductivity (EC) of substrate (dS⋅m^-1^)	3.3–5.1
(2) Moisture content of substrate (%)	56.8–70.2
(3) EC of nutrient solutions in the drainage tank (dS⋅m^-1^)	3.5–6.0
(4) Volume of nutrient solutions in the drainage tank (L)	2.1–9.8
(5) Cumulative drainage volume per day (L)	0–25.3
(6) EC of nutrient solutions in the mixing tank (dS⋅m^-1^)	2.1–2.9
(7) Volume of nutrient solutions in the mixing tank (L)	3.2–6.9
(8) Mixing volume of drainage (L)	0–3.3
(9) Mixing volume of water (L)	0–3.9
(10) Mixing volume of stock solution (L)	0–0.1
(11) Cumulative irrigation volume per day (L)	0–50.8
(12) Preset radiation integral for irrigation control (J⋅cm^-2^)	8.8–100.0
(13) Target volume of nutrient solutions per irrigation event per dripper (mL)	110.0–220.0
(14) CO_2_ concentration (μmol⋅mol^-1^)	312–574
(15) Light intensity (W⋅m^-2^)	0.0–293.3
(16) Temperature (°C)	16.5–33.8
(17) Relative humidity (%)	11.0–78.0
(18) Growth stage (day after transplanting, day)	99–176
(19) Plant height (cm)	115–181
(20) Number of nodes	18–31

### Recurrent Neural Network

**FIGURE 3:**
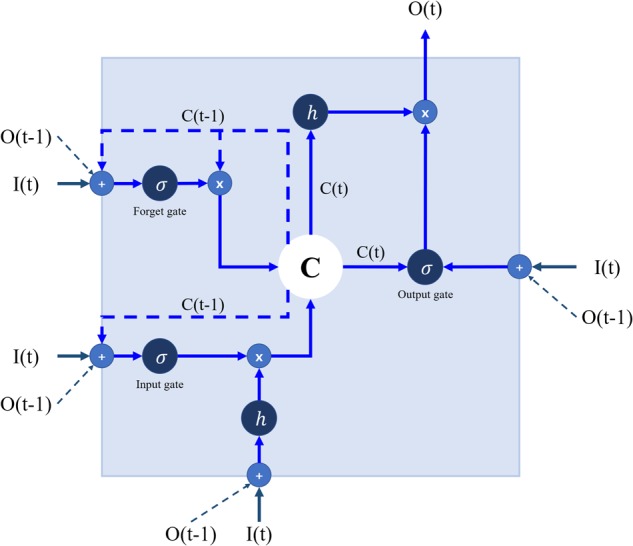
A structure of a long short-term memory (LSTM). I, input vectors; O, output vectors; C, cell state; *h, tanh* for input and output activation function; σ, sigmoidal function for gate activation function; *t* and *t*–1, current and previous times, respectively. Refer to **Table 1** for the input (I) and output (O).

Recurrent neural network algorithms deal with chronological data with a returning cycle. Long short-term memory (LSTM), an RNN algorithm, can solve the vanishing gradient problem of RNN (Hochreiter and Schmidhuber, 1997). This means that LSTM remembers the data of a long previous sequence. The core of LSTM algorithm is a cell with several gates (**Figure 3**). LSTM accepts previous data with addition operation, so vanishing gradient or exploding gradient problem is not occurred. Therefore, LSTM can analyze long time data than simple RNN.

Long short-term memory cells can retain, save, and load information about previous data. LSTM receives current input and previous output simultaneously, and the received information is operated through the gates. Previous information is saved as cell state, so sequenced data can be analyzed based on cell state. Gates are divided into three parts: input, forget, and output. The input gate determines how to select the data. The forget gate decides how much data should be forgotten and passes suitably forgotten previous data through a hyperbolic tangent function. The output gate combines cell state and input data and the combined output is sent to the next cell. The final output is printed when the predetermined time step is reached.

A modified LSTM algorithm called a gated recurrent unit (GRU) was developed (Cho et al., 2014). GRU has a similar structure to LSTM, except that it consists of update and reset gates. Since GRU has only two gates, it reduces computational complexity while retaining the advantages of LSTM. A specific RNN algorithm does not always yield the best prediction in all situations (Greff et al., 2015; Jozefowicz et al., 2015). Therefore, LSTM and GRU, the most well-known RNN algorithms, were compared. Similar to ordinary artificial neural networks (ANNs), RNN has hidden layers of perceptrons with activation function. In this study, input and output activation functions were set to hyperbolic tangent function, and gate activation function was set to sigmoidal function. The number of perceptrons and layers were variously combined to determine the optimal neural network structure.

Long short-term memory was adjusted to receive previous changes in environmental data and to predict the next hourly changes in substrate EC. The time step of LSTM was set every 6 h from 6 to 72 h and the output length set every 1 h from 1 to 24 h. The maximum time settings of output were used when comparing RNN structures. Then, input data were excluded one by one to determine which environment factors affect the change in root-zone EC.

To train the RNNs, the AdamOptimizer was used (Kingma and Ba, 2014). The hyperparameters for the LSTM and AdamOptimizer were set to commonly used values (**Table 2**). The GRU has the same hyperparameters as the AdamOptimizer, but forget bias does not need to be set. In the optimization process, neural networks are optimized to minimize cost (Rumelhart et al., 1988). In this study, mean square error (MSE) was used as a cost. Empirically, regressions based on ANNs usually use MSE instead of root-mean-square error (RMSE) as a cost for reducing computation (Esfe et al., 2016; Wang F. et al., 2017). The coefficient of determination (*R*^2^) was used for training and test accuracy. RMSE was also used for verifying model robustness. TensorFlow (v. 1.2.1, Python Deep Learning Library, Google, Menlo Park, CA, United States) was used for the experiments.

**Table 2:** Hyperparameters for recurrent neural network (RNN) and AdamOptimizer.

Parameter	Value	Description
Learning rate	0.001	Learning rate used by the AdamOptimizer
β_1_	0.9	Exponential mass decay rate for the momentum estimates
β_2_	0.999	Exponential velocity decay rate for the momentum estimates
E	1e-0.8	A constant for numerical stability
Forget bias^∗^	1.0	Probability of forgetting information in the previous dataset
Time step	2–24	Number of datasets that the LSTM will see at one time

*^∗^Forget bias was used only for long short-term memory (LSTM).*

### Data Preprocessing

Since RNNs use tangent functions and sigmoid functions internally, input data had to be normalized from 0 to 1 to improve training efficiency. In this study, RNN algorithms had the maximum time step of 72 h in environmental changes (input) and maximum output length of 24 h in EC changes (output). Both were trained after being combined into a single dataset. Data from 15 October to 24 December were randomly divided into training and validation datasets, and the rest of the data from December 25–31 were used to test the trained RNNs. Among the total datasets, 900 were used for training, 396 for validation, and 120 for test.

## Results

### Accuracy of the Trained Models

**FIGURE 4:**
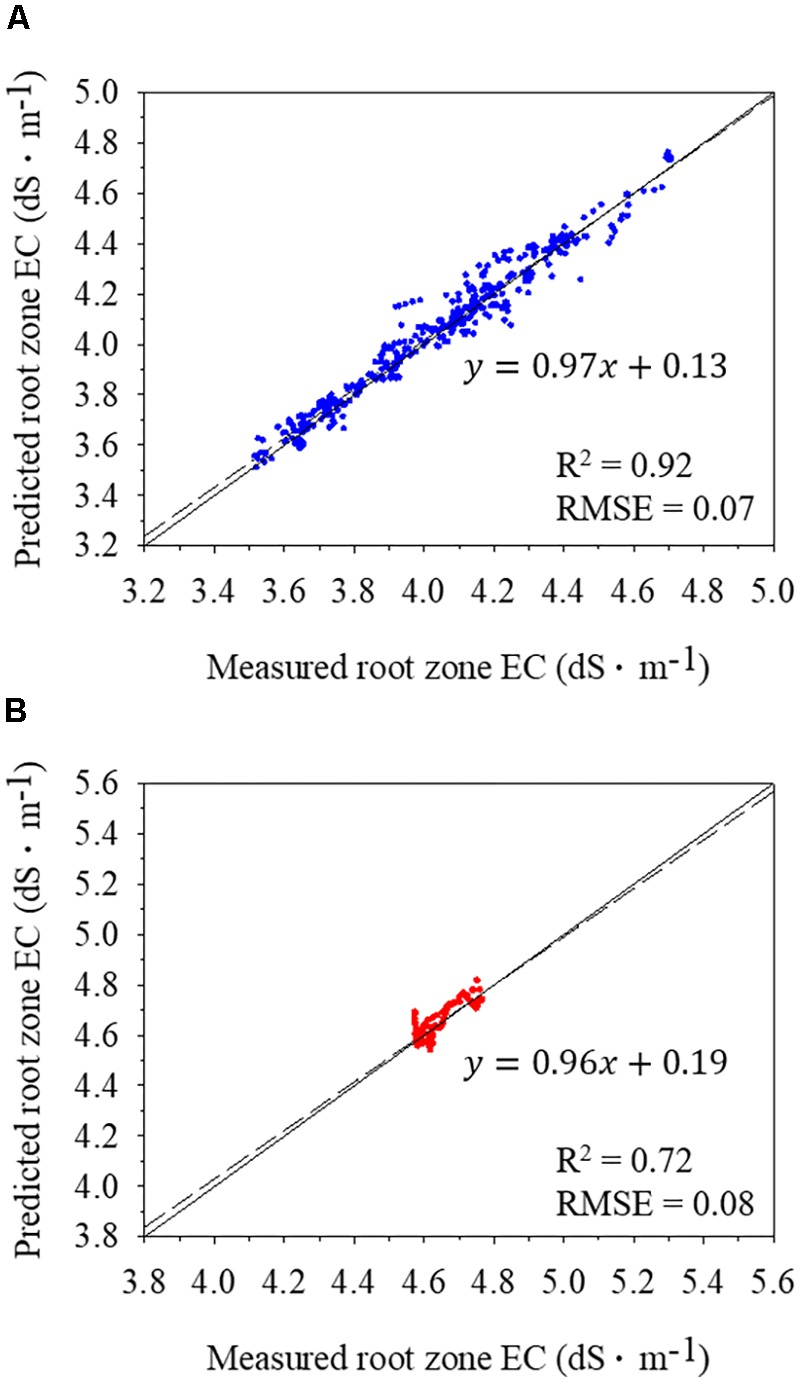
Comparisons of predicted and measured root-zone electrical conductivities (ECs) of nutrient solutions using validation **(A)** and test **(B)** datasets. Solid and dashed lines represent 1:1 line and regression line, respectively. The average of each 24-long output was presented.

Among all RNN structures, an LSTM of a single layer with 64 perceptrons showed the highest accuracy (**Table 3**). Although the RMSE of all structures ranged from 0.08 to 0.09, the single-layered LSTM showed the highest test accuracy with *R*^2^ = 0.72. Multi-layers did not improve the accuracy of RNN models. Regardless of the number of layers, LSTM showed the higher accuracy than GRU. For the same training condition, which had multi-inputs and -outputs, conventional algorithms such as ARIMA model, multivariate regression, or multi-layer perceptrons could not be trained. With the validation datasets, *R*^2^ was 0.92 and RMSE was 0.07 with the LSTM (**Figure 4A**), which was much higher than the test accuracy with *R*^2^ = 0.72 and RMSE = 0.08 (**Figure 4B**). Because each 24-long output was a result of one calculation, the average of each predicted and measured values was compared.

**Table 3:** Test accuracies and root mean square errors (RMSEs) of trained recurrent neural network (RNN) algorithms.

Type of RNN	Test accuracy (*R*^2^)	Test RMSE
Long short-term memory (LSTM)	0.72	0.08
Gated recurrent unit (GRU)	0.68	0.09
Multi-layered LSTM	0.70	0.08
Multi-layered GRU	0.68	0.09

### Optimization of Model Parameters

**FIGURE 5:**
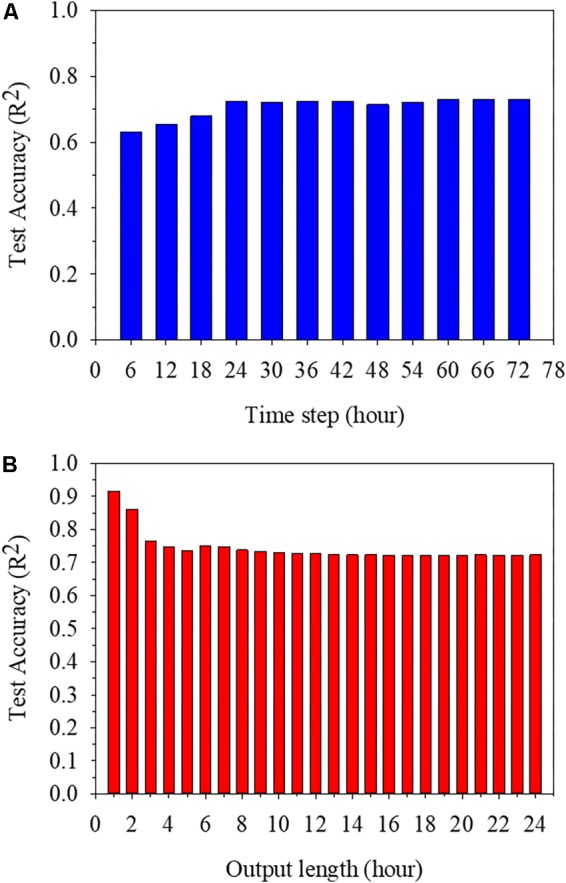
Test accuracies of trained long short-term memory (LSTM) algorithms at different time steps **(A)** and output lengths **(B)**.

The accuracy of LSTM tended to increase with extension of time step and reduction of output length. The accuracy of LSTM was highest when the time step was set to maximum. *R*^2^ for the test datasets was no less than 0.65 when the time step was longer than 12 h (**Figure 5A**). The time step longer than 24 h did not improve the accuracy. Meanwhile, *R*^2^ was lowest when output length was 24 h, but all *R*^2^s were no less than 0.72 (**Figure 5B**).

Among the input data, the EC of nutrient solutions in the drainage tank and cumulative irrigation volume per day most affected the substrate EC (**Table 4**). Both inputs reduced the test accuracy by 0.05. Substrate EC was the least influential factor in accuracy because the accuracy was rarely lowered even without substrate EC. For all inputs, the average value of reduction was 0.0295.

**Table 4:** Test accuracies of the long short-term memory (LSTM) after excluding input data.

Excluded data	Test accuracy (*R*^2^)	Excluded data	Test accuracy (*R*^2^)
(1)^z^	0.72	(11)	0.67
(2)	0.70	(12)	0.69
(3)	0.67	(13)	0.68
(4)	0.69	(14)	0.70
(5)	0.69	(15)	0.68
(6)	0.68	(16)	0.69
(7)	0.69	(17)	0.68
(8)	0.68	(18)	0.70
(9)	0.69	(19)	0.70
(10)	0.70	(20)	0.71

*^z^Refer to **Table 1** for the excluded data number.*

### Chronological Comparisons of Prediction

**FIGURE 6:**
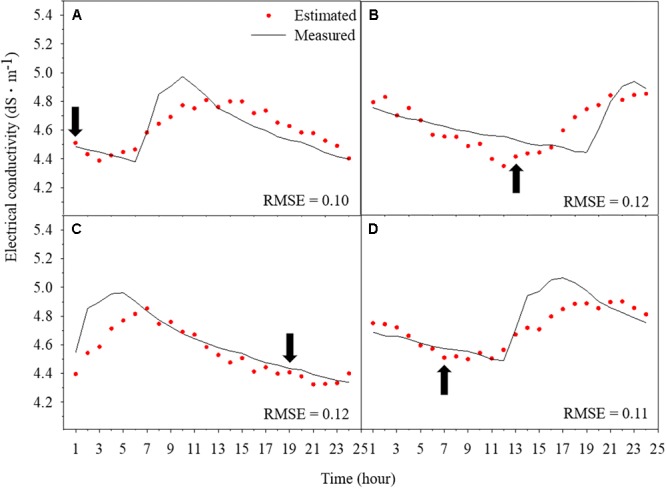
Chronological comparisons of predicted root-zone electrical conductivity (EC) via trained long short-term memory (LSTM) and measured data from 0:00 to 23:00 on December 25 **(A)**, from 6:00 on December 26 to 5:00 on December 27 **(B)**, from 12:00 on December 27 to 11:00 on December 28 **(C)**, and from 18:00 on December 29 to 17:00 on December 30 **(D)**. Arrows represent the point of 00:00.

**FIGURE 7:**
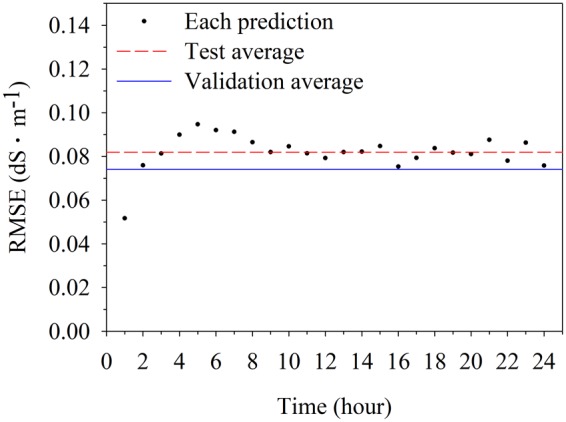
Root mean square errors (RMSEs) of electrical conductivity (EC) of nutrient solutions. RMSEs separately calculated based on each prediction for 1 h were compared with total validation and test RMSEs.

Trained LSTM detected the tendency and predicted the changes in EC, although there is little deviation between predicted and measured values (**Figure 6**). Prediction results followed the fluctuation of root-zone EC, even though variations from actual values occurred. The prediction of future 24-h EC showed different RMSEs (**Figure 7**). In particular, the first 3-h prediction showed lower RMSEs than the total validation RMSE. The RMSE tended to be higher in the data before 12-h, which was the beginning of the forecast. Especially, the RMSEs were lower in the first 3 h and became higher for 4–8 h. However, there was no large gap by time.

## Discussion

In this study, RNN showed the test *R*^2^ of 0.72, indicating that RNN had a possibility of predicting future tendency of EC changes (**Figure 4**). The trained RNNs with relatively shallow layers showed better accuracies in this study (**Table 3**). Recently, neural networks have deep structure in general (Ioffe and Szegedy, 2015; Silver et al., 2016) and RNNs have a very deep structure over time and therefore do not require fully connected multilayers in most cases (Jozefowicz et al., 2015). If the number of layers is the same, LSTM has more complex structure than GRU and has more parameters (Chung et al., 2014), resulting in higher accuracy. However, in case that the number of parameters should be small due to computational limitation, GRU can be used because the accuracy is not much different. Although ARIMA model is an algorithm to analyze chronological data, it could not predict future substrate EC. Since ARIMA model uses only target factor changes as input, it seems that the change in substrate EC itself did not show a definite periodicity. On the other hand, RNN can use other environmental factors as input, so it can correlate environmental changes with root-zone EC changes. Moreover, RNN has a unique structure and deals with huge sizes of input and output, so it is difficult to compare RNN with conventional algorithms or models.

Considering the accuracies in recent deep learning applications, the test *R*^2^ of 0.72 in this study is not high and would be due to the relatively short estimation period (**Figure 4**). The period was a fraction of the cropping season and the data used for prediction was from 99 days after transplanting. Therefore, the earlier age of the plants could not be used because it was out of the trained ranges. However, deep learning algorithms can be more accurate when tested with big data of long periods to generalize to all possible conditions (Lopez et al., 2001). Other agricultural studies using deep learning have been conducted with big datasets with long collecting periods to cover almost all possibilities, such as seasonal influences (Trejo-Perea et al., 2009; Wang H. et al., 2017). Therefore, all datasets of other periods could improve model robustness. Adding more environmental and plant growth data to input elements can also increase the accuracy. Virtual conditions via simulation could be helpful for training the neural network (Beltramo et al., 2016). Moreover, if data are collected by similar methods used in this study, the trained LSTM can be applied to other periods or other plants using transfer learning (Gao et al., 2014; Shin et al., 2016).

EC interacts with crops and ambient environments continuously, so previous environments are related with EC changes. Therefore, the accuracy was improved with increasing time step, which represents the length of previous information (**Figure 5A**). However, since the nutrient solution was closed and controlled, the previous information more than 24 h did not affect the prediction accuracy. Therefore, 24-h time step is an appropriate input length. Meanwhile, the accuracy was deteriorated with increasing output lengths due to the increase in computation (**Figure 5B**). Obviously, the accuracy was better because the values to be predicted were reduced when the output length was shorter. However, output lengths that are too short cannot be used to help control nutrient solutions through EC forecasting. EC should be predictable from sunrise, at least when transpiration begins because transpiration and water have a significant interaction (Kramer, 1937; Greenwood and Beresford, 1979). Therefore, the maximum output length should be selected to predict the hourly changing tendency of EC in a day by using changes in environmental factors from the morning and previous day.

Through excluding input elements, it was found that the drainage nutrient solutions were highly related with the substrate EC (**Table 4**). However, the accuracy was not changed even if the substrate EC was eliminated from the input elements. LSTM uses the cell state to transmit the information of previous output (Greff et al., 2015; Heffernan et al., 2017). The information about the substrate EC, which is the output, could be included in the cell state. However, the accuracy was reduced by excluding water-related environments in **Table 1**. The changes in EC are also affected by water content (Rhoades et al., 1976; Medrano et al., 2005). Therefore, water-related data were important to predict the substrate EC. However, it can be said that all input factors were appropriate because the accuracy does not collapse by exclusion of certain factors.

The difference between accuracies resulted from generalizing the entire range of data (**Figure 6**). Predicting EC changes during the day was difficult because the plants disturbed the water and nutrient environments by transpiration. Underestimated or overestimated predictions between about 6 and 12 a.m. could be resulted from the increasing transpiration. In addition, transpiration significantly affects the uptake of nutrients, which is related with the change in root-zone EC and varies with growth stage (Van Noordwijk, 1990; Baille et al., 1994; Le Bot et al., 1998). Therefore, variation of the root-zone EC can be larger depending on growth stage even when the drainage rate is controlled (Massa et al., 2011; Shin and Son, 2016). In the study, the data were acquired in the latter part of the cultivation (**Table 1**). Since the crops were sufficiently grown, a relatively large change in the substrate EC was observed. Therefore, the trained LSTM showed a low test accuracy, but it was acceptable performance.

The RMSE showed that the trained LSTM was able to predict the entire range with even accuracy (**Figure 7**). Due to the nature of LSTM, which is a black box modeling, it is impossible to understand exactly what affected the RMSEs. It would not be the effect of EC change at a specific time slot because the model predicted the substrate EC at 10-min intervals. Further studies about RNN structure are needed to reveal the reason of slight differences in accuracy. However, the principle of EC-based nutrient control is maintaining the EC of nutrient solutions at a set point (Ahn et al., 2010). Therefore, predicting whether the EC will increase or decrease in the future can help with sophisticated nutrient control. Because nutrient solution control depends on a contemporary EC in current soilless cultures (Neto et al., 2014; Kinoshita et al., 2016), predicted 3-h EC might improve the accuracy of nutrient control. In addition, since the RMSE did not change much near the test accuracy of 0.08 after the 9 h, it can be said that stable forecasts during a day are possible.

Comprehensively, LSTM showed acceptable accuracies in predicting substrate EC. In addition, it is known that EC and pH can be predicted together using ANNs (Ferentinos and Albright, 2002). In this study, the pH data were not used for model training, but the pH of nutrient solution could be predicted using the LSTM. Therefore, if pH and EC can be predicted together, growers could be able to cope with rapid changes in nutrient concentration caused by environmental changes. Furthermore, LSTM, which is effective in analyzing chronological data, could predict plant environments influenced by the accumulations of previous situations, such as plant growth and ion concentration of nutrient solutions.

## Conclusion

Prediction models used in this study were based on a deep learning algorithm, RNN. Among the most popular RNN algorithms, a single-layered LSTM showed the highest test accuracy (*R*^2^ = 0.72). The trained LSTM could be applied to control nutrient solutions in closed-loop soilless cultures based on prediction of future EC. Therefore, the algorithm could make planned management of nutrient solutions possible, reducing resource wastes. Prediction accuracy could be higher with additional data. Deep learning algorithms could be more accurate with additional data, so other environmental factors or plant growth data could improve model robustness. In particular, the LSTM can be extended to predict various factors which are influenced by the accumulations of previous situations. Further research on long-period control using LSTM is required.

## Author Contributions

TM constructed the artificial neural network, analyzed the root-zone EC, and wrote the manuscript. TIA developed the open-loop soilless culture system and measured the environment and growth data. JES designed and supervised the experiment and wrote the manuscript.

## Conflict of Interest Statement

The authors declare that the research was conducted in the absence of any commercial or financial relationships that could be construed as a potential conflict of interest. The reviewer AC and the handling editor declared their shared affiliation.
